# Crystal structure of 4-(pyrazin-2-yl)morpholine

**DOI:** 10.1107/S2056989018000312

**Published:** 2018-01-12

**Authors:** Siva Sankar Murthy Bandaru, Anant Ramakant Kapdi, Carola Schulzke

**Affiliations:** a Institut für Biochemie, Ernst-Moritz-Arndt Universität Greifswald, Felix-Hausdorff-Strasse 4, D-17487 Greifswald, Germany; b Department of Chemistry, Institute of Chemical Technology, Nathalal Parekh Road, Matunga, Mumbai 400 019, India

**Keywords:** crystal structure, palladium-catalysed reactions, Buchwald–Hartwig amination, pyrazine, morpholine

## Abstract

The mol­ecular structure of the title compound, C_8_H_11_N_3_O, is nearly planar despite the chair conformation of the morpholine moiety. In the crystal, the mol­ecules form sheets parallel to the *b* axis, which are supported by non-classical hydrogen-bonding inter­actions between C—H functionalities and the O atom of morpholine and the 4-N atom of pyrazine, respectively. The title compound crystallizes in the monoclinic space group *P*2_1_/*c* with four mol­ecules in the unit cell.

## Abstract 2

The N atom of morpholine was coupled to the 2-carbon atom of pyrazine in a Pd^II^/phosphatri­aza­adamantyl butane saltone-catalysed reaction and crystallized from the eluent (EtOAc–hexa­ne) after chromatography. In the crystal, the mol­ecules form sheets parallel to the *b* axis, which are supported by non-classical hydrogen-bonding inter­actions between C—H functionalities and the O atom of morpholine and the 4-N atom of pyrazine, respectively.

## Chemical context   

The potential applications of aryl and heteroaryl amines in chemistry, materials science and pharmaceutical industries encourages research into the formation of C—N bonds (Rappoport, 2007 ▸; Lawrence, 2004 ▸, Weissermel & Arpe 1997 ▸). *N*-Hetero­aryl­morpholine moieties are prevalent in biologically active mol­ecules such as medicines for the treatment of schizophrenia or type-2 diabetes mellitus (Bartolomé-Nebreda *et al.*, 2014 ▸). In this context we are engaged in the synthesis of a library of heterocyclic amine derivatives. In course of these investigations, pure crystalline 4-(pyrazin-2-yl)morpholine was isolated with the crystals being obtained upon purification by column chromatography.
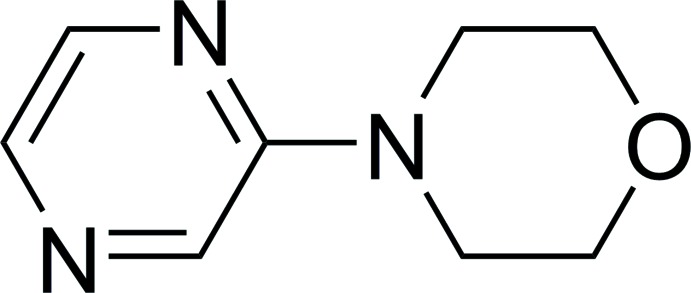


## Structural commentary   

**Figure 1:**
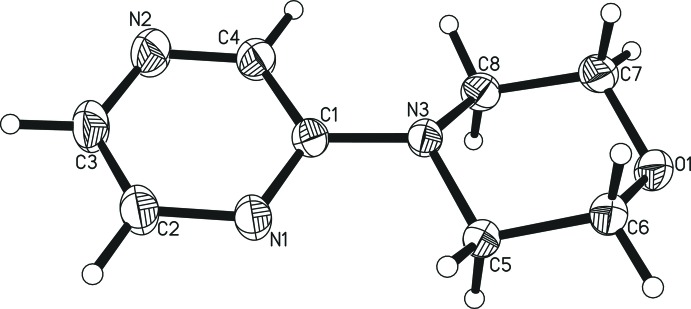
The mol­ecular structure of the title compound, showing the atom labelling and 50% probability displacement ellipsoids.

4-(Pyrazin-2-yl)morpholine (Fig. 1 ▸) crystallizes in the monoclinic space group *P*2_1_/*c* with four mol­ecules in the unit cell. There are reports in the literature of the mol­ecular structures of compounds in which the morpholine nitro­gen atom is coupled to the carbon atom of a non-annelated *N*-heterocyclic pyridine (Dahlgren *et al.*, 2012 ▸; Horton *et al.*, 2012 ▸; Huth *et al.*, 2007 ▸; Klauschenz *et al.*, 1994 ▸; Li *et al.*, 2014 ▸, Reck *et al.*, 1992 ▸) or pyrimidine (Cheprakova *et al.*, 2014 ▸; García *et al.*, 2009 ▸; Gorbunov *et al.*, 2013 ▸; Hansen & Geffken, 2012 ▸; Vinogradova *et al.*, 2016 ▸). For pyrazine as the heterocycle, however, (to the best of our knowledge and after conducting a database search, see §4) the present work constitutes the first structural report even though the title compound itself has been known since 1969 (Abe *et al.*, 1969 ▸).

The orientation of the morpholine ring, in its typical chair conformation, relative to the aromatic plane can be either more or less in plane (*e.g.* Vinogradova *et al.*, 2016 ▸), tilted around the N—C bond *(e.g.* Li *et al.*, 2014 ▸), bent away from the aromatic plane (*e.g.* Hansen & Geffken, 2012 ▸) or a combination of the latter two (*e.g.* Reck *et al.*, 1992 ▸), depending on the other substituents on the heterocycle. In the present case, a morpholine ring is as much aligned with the N1/N2/C1–C4 plane as its conformation allows, with the carbon C8 showing the largest distance from the plane of 0.414 (1) Å. This distance is shorter than for any of the pyridine or pyrimidine derivatives without morpholine disorder from the reports mentioned above. The largest deviation from the plane of the pyrizine atoms was found to be 0.013 (1) Å for C1 and C4.

The quality of the crystallographic data allowed the hydrogen atoms to be located and refined entirely freely without any constraints or restraints. The information content of the metrical parameters involving the hydrogen atoms, including non-classical hydrogen-bonding inter­actions, is therefore comparably high. The C—H distances for the aromatic atoms are 0.999 (15) Å for C2, 0.976 (16) Å for C3 and 0.962 (16) Å for C4. The methyl­ene protons are in a distance range from their parent carbon atoms of 0.978 (14) to 1.016 (14) Å with a tendency for the longer C—H bond to be for the hydrogen atom in the axial position [only C7 is an exception with distances of 1.003 (14) Å for the axial and 1.005 (14) Å for the equatorial position]. All C—C, C—N and C—O bond lengths are within the commonly observed ranges.

## Supra­molecular features   

**Figure 2:**
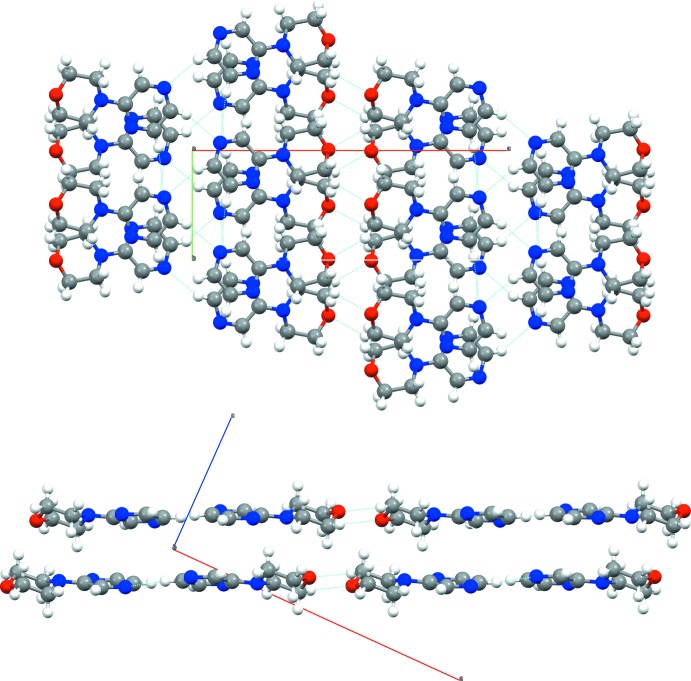
The crystal packing (*Mercury*; Macrae *et al.*, 2006 ▸) viewed (top) along the *c* axis and (bottom) along the *b* axis showing the layered arrangement and the non-classical hydrogen-bonding inter­actions (Table 1 ▸) between the mol­ecules of a sheet.

In the crystal, the mol­ecules form sheets parallel to the *b* axis supported by non-classical hydrogen-bonding inter­actions (Fig. 2 ▸, Table 1 ▸). In each mol­ecule, the pyrazine ring is tilted slightly out of the general orientation of the sheets and the direction of the rotation alternates between adjacent rows (protruding along the *b* axis) as well as between adjacent layers with an angle of 17.95° between the two variants of torsion.

Within the sheets, each mol­ecule forms hydrogen-bonding interactions to six surrounding mol­ecules. These are donor inter­actions involving C2 [C2—H2⋯N2(*x*, *y* + 1, *z*)], C3 [C3—H3⋯N2(−*x* + 2, *y* + 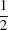, −*z* + 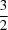], C4 [C4—H4⋯N1(*x*, *y* − 1, *z*)] and C6 [C6—H6*A*⋯O1(−*x* + 1, *y* + 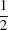, −*z* + 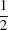)] and acceptor inter­actions involving N1 [N1⋯H4—C4(*x*, *y* + 1, *z*)], N2 [N2⋯H2—C2(*x*, *y* − 1, *z*), N2⋯H3—C3(−*x* + 2, *y* + 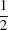, −*z* + 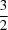] and O1 [O1⋯H6*A*—C6(1 − *x*, 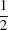 + *y*, 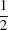 − *z*)].

No π–π inter­actions are apparent between the sheets, with the closest distance between aromatic ring centroids being 4.2470 (11) Å while two sheets are 3.564 Å apart.

## Synthesis and crystallization   

The synthesis was carried out under an inert gas atmosphere (N_2_) applying the typical Schlenk line procedures. To an oven-dried Schlenk tube (25 mL) were added Pd(OAc)_2_ (1 mol%, 0.0024 g) and PTABS (phosphatriazene adamantyl butane saltone; 2 mol%, 0.00586 g) and a nitro­gen atmosphere was generated. To this were added 3 mL of dry DMF followed by the addition of 2-chloro­pyrazine (0.086 mL, 1mmol), 1.5 equivalents of tri­ethyl­amine (0.3 mL, 1.5 mmol) and 1.1 equivalent of morpholine (0.1 mL, 1.1 mmol). The suspension was stirred at room temperature for 4 h and progress of the reaction was monitored by TLC. After completion of the reaction, the crude product was purified and isolated by column chromatography in an EtOAc:hexane (1:3) solvent system. The final sharp colourless needles (0.124 mg, 0.83 mmol, 83%) were obtained directly after the column purification step by crystallizing from the eluent. The mounted crystal was a block cut from a large needle. The compound has a low melting point of only 318 K and the crystals were stored in the fridge until they were measured.

^1^H NMR (300 MHz, chloro­form-*d*) δ ppm 3.51–3.63 (*m*, 4 H), 3.79–3.90 (*m*, 4 H), 7.90 (*d*, *J* = 2.64 Hz, 1 H), 8.14 (*d*, *J* = 7.6Hz, 1 H), 9.61 (*d*, *J* = 7.8 Hz, 1 H). ^13^C NMR (75 MHz, chloro­form-*d*) δ ppm 45.18 (*s*, 1C) 66.93 (*s*, 1C) 77.42 (*s*, 1C) 77.84 (*s*, 1C) 131.33 (*s*, 1C) 133.98 (*s*, 1C) 142.16 (*s*, 1C) 155.48 (*s*, 1C). ESI–MS (*m*/*z*) = 166.17 (*M* + H)^+^, 167.22 (*M* + 2H)^2+^ (*cf*. Graham *et al.*, 2011 ▸).

## Refinement   

Crystal data, data collection and structure refinement details are summarized in Table 2 ▸. All hydrogen atoms were located and refined freely without any constraints or restraints.

## Supplementary Material

Crystal structure: contains datablock(s) I. DOI: 10.1107/S2056989018000312/ds2249sup1.cif

Supporting information file. DOI: 10.1107/S2056989018000312/ds2249Isup2.cml

CCDC reference: 1585321

Additional supporting information:  crystallographic information; 3D view; checkCIF report

## Figures and Tables

**Table 1:** Hydrogen-bond geometry (Å, °)

*D*—H⋯*A*	*D*—H	H⋯*A*	*D*⋯*A*	*D*—H⋯*A*
C6—H6*A*⋯O1^i^	0.988 (14)	2.561 (14)	3.4841 (16)	155.5 (10)
C3—H3⋯N2^ii^	0.976 (16)	2.670 (16)	3.5723 (19)	153.9 (13)
C2—H2⋯N2^iii^	0.999 (15)	2.743 (15)	3.6840 (19)	157.2 (11)
C4—H4⋯N1^iv^	0.962 (16)	2.787 (16)	3.6775 (18)	154.3 (11)

Symmetry codes: (i) 
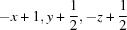
; (ii) 
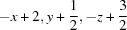
; (iii) 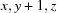; (iv) 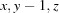.

**Table 2:** Experimental details

Crystal data	
Chemical formula	C_8_H_11_N_3_O
*M*_r_	165.20
Crystal system, space group	Monoclinic, *P*2_1_/*c*
Temperature (K)	170
*a*, *b*, *c* (Å)	17.069 (3), 5.9278 (12), 7.8053 (16)
β (°)	90.54 (3)
*V* (Å^3^)	789.7 (3)
*Z*	4
Radiation type	Mo *K*α
μ (mm^−1^)	0.10
Crystal size (mm)	0.38 × 0.31 × 0.26

Data collection	
Diffractometer	Stoe IPDS2T
No. of measured, independent and observed [*I* > 2σ(*I*)] reflections	6583, 1666, 1285
*R*_int_	0.044
(sin θ/λ)_max_ (Å^−1^)	0.634

Refinement	
*R*[*F* ^2^ > 2σ(*F* ^2^)], *wR*(*F* ^2^), *S*	0.032, 0.081, 1.01
No. of reflections	1666
No. of parameters	153
H-atom treatment	All H-atom parameters refined
Δρ_max_, Δρ_min_ (e Å^−3^)	0.19, −0.18

Computer programs: *X-AREA* (Stoe & Cie, 2010 ▸), *SHELXT2016* (Sheldrick, 2015*a* ▸), *SHELXL2016* (Sheldrick, 2015*b* ▸), *XP* in *SHELXTL* (Sheldrick, 2008 ▸), *Mercury* (Macrae *et al.*, 2006 ▸) and *CIFTAB* (Sheldrick, 2008 ▸).

## supplementary crystallographic information

### Crystal data

**Table:**

C_8_H_11_N_3_O	*F*(000) = 352
*M**_r_* = 165.20	*D*_x_ = 1.389 Mg m^−^^3^
Monoclinic, *P*2_1_/*c*	Mo *K*α radiation, λ = 0.71073 Å
*a* = 17.069 (3) Å	Cell parameters from 7070 reflections
*b* = 5.9278 (12) Å	θ = 5.2–53.6°
*c* = 7.8053 (16) Å	µ = 0.10 mm^−^^1^
β = 90.54 (3)°	*T* = 170 K
*V* = 789.7 (3) Å^3^	Block, colourless
*Z* = 4	0.38 × 0.31 × 0.26 mm

### Data collection

**Table:**

Stoe IPDS2T diffractometer	1285 reflections with *I* > 2σ(*I*)
Radiation source: fine-focus sealed tube	*R*_int_ = 0.044
Detector resolution: 6.67 pixels mm^-1^	θ_max_ = 26.8°, θ_min_ = 3.6°
ω scans	*h* = −21→21
6583 measured reflections	*k* = −7→7
1666 independent reflections	*l* = −9→9

### Refinement

**Table:**

Refinement on *F*^2^	0 restraints
Least-squares matrix: full	Hydrogen site location: difference Fourier map
*R*[*F*^2^ > 2σ(*F*^2^)] = 0.032	All H-atom parameters refined
*wR*(*F*^2^) = 0.081	*w* = 1/[σ^2^(*F*_o_^2^) + (0.0391*P*)^2^ + 0.1242*P*] where *P* = (*F*_o_^2^ + 2*F*_c_^2^)/3
*S* = 1.01	(Δ/σ)_max_ < 0.001
1666 reflections	Δρ_max_ = 0.19 e Å^−^^3^
153 parameters	Δρ_min_ = −0.18 e Å^−^^3^

### Special details

**Table:**

Geometry. All esds (except the esd in the dihedral angle between two l.s. planes) are estimated using the full covariance matrix. The cell esds are taken into account individually in the estimation of esds in distances, angles and torsion angles; correlations between esds in cell parameters are only used when they are defined by crystal symmetry. An approximate (isotropic) treatment of cell esds is used for estimating esds involving l.s. planes.

### Fractional atomic coordinates and isotropic or equivalent isotropic displacement parameters (Å^2^)

**Table:**

	*x*	*y*	*z*	*U*_iso_*/*U*_eq_	
O1	0.56805 (5)	0.49320 (14)	0.28629 (10)	0.0288 (2)	
N1	0.80725 (6)	0.78798 (17)	0.57424 (13)	0.0273 (2)	
N2	0.91130 (6)	0.4299 (2)	0.62453 (16)	0.0389 (3)	
N3	0.71160 (5)	0.53996 (16)	0.47126 (12)	0.0225 (2)	
C1	0.78753 (7)	0.57747 (19)	0.53119 (14)	0.0227 (3)	
C2	0.87897 (7)	0.8160 (2)	0.64356 (18)	0.0327 (3)	
C3	0.93002 (8)	0.6409 (2)	0.66999 (19)	0.0370 (3)	
C4	0.84119 (7)	0.4000 (2)	0.55398 (17)	0.0318 (3)	
C5	0.66501 (6)	0.73760 (19)	0.42267 (15)	0.0226 (3)	
C6	0.57955 (7)	0.6729 (2)	0.40626 (15)	0.0255 (3)	
C7	0.61043 (7)	0.3001 (2)	0.34360 (16)	0.0271 (3)	
C8	0.69742 (7)	0.3467 (2)	0.35819 (15)	0.0258 (3)	
H6B	0.5600 (7)	0.621 (2)	0.5203 (17)	0.028 (3)*	
H5B	0.6834 (7)	0.804 (2)	0.3125 (16)	0.023 (3)*	
H5A	0.6694 (7)	0.853 (2)	0.5118 (17)	0.027 (3)*	
H8B	0.7235 (8)	0.210 (3)	0.4052 (17)	0.031 (4)*	
H7B	0.5899 (7)	0.248 (2)	0.4570 (17)	0.029 (3)*	
H6A	0.5487 (8)	0.802 (2)	0.3624 (16)	0.028 (3)*	
H4	0.8289 (8)	0.248 (3)	0.5193 (18)	0.038 (4)*	
H7A	0.6002 (8)	0.177 (2)	0.2579 (17)	0.028 (3)*	
H8A	0.7193 (8)	0.380 (2)	0.2402 (17)	0.034 (4)*	
H2	0.8931 (8)	0.975 (3)	0.6735 (17)	0.035 (4)*	
H3	0.9810 (9)	0.670 (3)	0.723 (2)	0.049 (4)*	

### Atomic displacement parameters (Å^2^)

**Table:**

	*U*^11^	*U*^22^	*U*^33^	*U*^12^	*U*^13^	*U*^23^
O1	0.0287 (5)	0.0263 (5)	0.0310 (4)	−0.0026 (4)	−0.0104 (3)	0.0010 (3)
N1	0.0217 (5)	0.0252 (5)	0.0348 (6)	−0.0007 (4)	−0.0041 (4)	−0.0036 (4)
N2	0.0255 (6)	0.0337 (6)	0.0571 (7)	0.0040 (5)	−0.0100 (5)	0.0021 (5)
N3	0.0200 (5)	0.0191 (5)	0.0283 (5)	0.0005 (4)	−0.0037 (4)	−0.0012 (4)
C1	0.0199 (5)	0.0240 (6)	0.0242 (5)	−0.0006 (4)	0.0009 (4)	0.0009 (4)
C2	0.0251 (6)	0.0303 (7)	0.0427 (7)	−0.0023 (5)	−0.0065 (5)	−0.0042 (6)
C3	0.0219 (6)	0.0382 (8)	0.0507 (8)	−0.0016 (6)	−0.0094 (6)	−0.0013 (6)
C4	0.0259 (6)	0.0247 (6)	0.0448 (7)	0.0013 (5)	−0.0057 (5)	0.0011 (5)
C5	0.0215 (6)	0.0212 (5)	0.0251 (6)	0.0003 (5)	−0.0023 (4)	0.0003 (5)
C6	0.0222 (6)	0.0245 (6)	0.0297 (6)	−0.0005 (5)	−0.0039 (5)	0.0006 (5)
C7	0.0300 (6)	0.0228 (6)	0.0282 (6)	−0.0044 (5)	−0.0054 (5)	0.0005 (5)
C8	0.0286 (6)	0.0209 (6)	0.0279 (6)	0.0001 (5)	−0.0024 (5)	−0.0023 (5)

### Geometric parameters (Å, º)

**Table:**

O1—C7	1.4238 (15)	C3—H3	0.976 (16)
O1—C6	1.4307 (14)	C4—H4	0.962 (16)
N1—C1	1.3345 (15)	C5—C6	1.5126 (16)
N1—C2	1.3441 (16)	C5—H5B	0.999 (13)
N2—C4	1.3244 (16)	C5—H5A	0.978 (14)
N2—C3	1.3381 (18)	C6—H6B	1.002 (13)
N3—C1	1.3917 (15)	C6—H6A	0.988 (14)
N3—C5	1.4639 (15)	C7—C8	1.5135 (17)
N3—C8	1.4648 (14)	C7—H7B	1.003 (14)
C1—C4	1.4051 (17)	C7—H7A	1.005 (14)
C2—C3	1.3693 (19)	C8—H8B	0.995 (15)
C2—H2	0.999 (15)	C8—H8A	1.016 (14)
			
C7—O1—C6	109.07 (9)	N3—C5—H5A	109.7 (8)
C1—N1—C2	116.31 (10)	C6—C5—H5A	107.7 (8)
C4—N2—C3	116.61 (11)	H5B—C5—H5A	108.3 (10)
C1—N3—C5	117.46 (9)	O1—C6—C5	111.74 (10)
C1—N3—C8	118.39 (9)	O1—C6—H6B	108.0 (7)
C5—N3—C8	112.55 (9)	C5—C6—H6B	109.3 (7)
N1—C1—N3	117.76 (10)	O1—C6—H6A	106.3 (7)
N1—C1—C4	120.38 (11)	C5—C6—H6A	110.1 (8)
N3—C1—C4	121.79 (11)	H6B—C6—H6A	111.4 (10)
N1—C2—C3	122.88 (12)	O1—C7—C8	111.85 (10)
N1—C2—H2	115.3 (8)	O1—C7—H7B	110.0 (8)
C3—C2—H2	121.9 (8)	C8—C7—H7B	109.9 (7)
N2—C3—C2	121.23 (12)	O1—C7—H7A	106.9 (7)
N2—C3—H3	119.1 (10)	C8—C7—H7A	110.3 (8)
C2—C3—H3	119.7 (10)	H7B—C7—H7A	107.8 (11)
N2—C4—C1	122.53 (12)	N3—C8—C7	110.14 (10)
N2—C4—H4	115.9 (9)	N3—C8—H8B	110.2 (8)
C1—C4—H4	121.6 (9)	C7—C8—H8B	108.3 (8)
N3—C5—C6	109.88 (10)	N3—C8—H8A	109.6 (8)
N3—C5—H5B	111.4 (7)	C7—C8—H8A	109.6 (8)
C6—C5—H5B	109.7 (7)	H8B—C8—H8A	108.9 (11)

### Hydrogen-bond geometry (Å, º)

**Table:**

*D*—H···*A*	*D*—H	H···*A*	*D*···*A*	*D*—H···*A*
C6—H6*A*···O1^i^	0.988 (14)	2.561 (14)	3.4841 (16)	155.5 (10)
C3—H3···N2^ii^	0.976 (16)	2.670 (16)	3.5723 (19)	153.9 (13)
C2—H2···N2^iii^	0.999 (15)	2.743 (15)	3.6840 (19)	157.2 (11)
C4—H4···N1^iv^	0.962 (16)	2.787 (16)	3.6775 (18)	154.3 (11)

Symmetry codes: (i) −*x*+1, *y*+1/2, −*z*+1/2; (ii) −*x*+2, *y*+1/2, −*z*+3/2; (iii) *x*, *y*+1, *z*; (iv) *x*, *y*−1, *z*.
